# Knowledge, Perceptions, Challenges and opportunities in achieving sustainable coverage of mass drug administration towards the control and elimination of Schistosomiasis and Soil Transmitted Helminths in hard-to-reach communities of Ghana

**DOI:** 10.1371/journal.pntd.0012664

**Published:** 2024-11-21

**Authors:** Akua Obeng Forson, Patrick F. Ayeh-Kumi, Abdul Rahim Mohammed, Isaac Kwame Sraku, Gustavus Adolphus Myers-Hansen, Yaw Asare Afrane

**Affiliations:** 1 Centre for Vector-borne Diseases Research, Department of Medical Microbiology, University of Ghana Medical School, University of Ghana, Korle-Bu, Accra, Ghana; 2 Department of Medical Laboratory Science, School of Biomedical and Allied Health Sciences, University of Ghana, Korle-Bu, Accra, Ghana; 3 Department of Biological Environmental and Occupational Health Sciences, School of Public Health, University of Ghana, Legon, Accra, Ghana; Chung-Ang University, REPUBLIC OF KOREA

## Abstract

**Introduction:**

In Ghana, schistosomiasis (SCH) and soil-transmitted helminths (STH) infections are of major public health problems in children. In the last decades, various interventions have been instituted by the Ghana Health Service (GHS) in collaboration with non-governmental organizations (NGOs) for the control and subsequent elimination of SCH and STH. However, these infections still remain common in both adults and children in many districts in Ghana. This study aimed to identify challenges in achieving sustainable coverage of mass drug administration for the control of STH and SCH and to explore opportunities to scale up its implementation among people living in hard-to-reach communities.

**Method:**

Twelve focus group discussions (FGDs) with community members were conducted to access challenges to mass drug administration (MDA), and 20 in-depth interviews (IDIs) with key informants were conducted to access opportunities to optimize MDA to control and eliminate soil-transmitted helminth infections and schistosomiasis in hard-to-reach communities.

**Results:**

Results showed participants held the correct notions of SCH and STH, and expressed willingness to participate in the MDA program. However, the lack of community drug distributors (CDDs) in the communities, inadequate and misleading information of MDA activities, and general concern about the adverse effects of MDA medications were some of the challenges identified to hinder MDA operations.

**Conclusion:**

Transitioning from SCH and STH control to elimination goals requires intensive health education campaigns before MDA are conducted in hard-to-reach communities in Ghana. Furthermore, there is a need for political members and policymakers to collaborate in providing scarce and sanitary infrastructure and continuously provide disease-specific information to community members to address and dispel common misconceptions and anxieties regarding the transmission and treatment of SCH and STH.

## Author summary

In Ghana, schistosomiasis (SCH) and soil-transmitted helminths (STH) infections are **of major public health problems in children**. The aim of this study was to **identify challenges in achieving sustainable coverage of mass drug administration (MDA) for the control of STH and SCH,** and to investigate potential avenues for expanding its use among the populace residing in **hard-to-reach communities**. Focus group discussions (FGDs) with community members and in-depth interviews (IDIs) with key informants were held to address these aims. The study revealed the participants **expressed willingness to participate in the MDA program**, however, lack of community drug distributors (CDDs), **inadequate and misleading information on MDA activities**, and widespread worry about the negative effects of MDA drugs were major challenges **identified. Transitioning from SCH and STH control to elimination goals requires** extensive health education, and politicians and policymakers must work together to provide scarce and sanitary infrastructure in endemic communities.

## Introduction

In 2022, 1.63 billion people required interventions against NTDs, with the highest proportion in South-East Asia (51.4%), and Sub-Saharan Africa (35.8%) [1]. In Ghana, the endemicity of NTDs such as lymphatic filariasis, onchocerciasis, schistosomiasis and soil-transmitted helminths is reported to overlap geographically in many districts in Ghana [2]. Schistosomiasis (SCH) caused by the blood fluke *Schistosoma haematobium* is widespread in Ghana and the intestinal schistosomiasis caused by *S*. *mansoni* is restricted to some districts in Ghana [2]. Nationwide mapping of schistosomiasis in 2008 revealed varied prevalence with 48 districts classified as highly (≥ 50%) endemic and 137 districts classified as moderately (10% - 50%) endemic with a total of 6,618,064 school aged children at risk of infections. Generally, communities along the Volta basin in Ghana have been reported to have prevalences of 80–90% for SCH [3]. However, some studies have likewise reported prevalence of 3.3% - 48.5% for *S*. *haematobium* and 30% - 78.3% for *S*. *mansoni in* children and adults in Accra [4–6]. The nationwide mapping in 2008 for soil-transmitted helminthiasis (STH) reported high prevalence of STH in 17 districts of Ghana, with 357,203 school-aged population at risk of infections [7]. A recent study by Akosah-Brempong *et al*., [8] from Island communities along the Volta Lake in one of the districts reported SCH prevalence of >70% and between 5–7% for STHs in school children [8]. The study also reported higher SCH and STH infection rates in children in hard-to-reach communities compared to those living on the mainland [8]. In addition, varying prevalence of STH have been reported in children (12–59%) [9–11], pregnant women (4% - 53%) [12,13], and in adults engaged in farming or food retailing (5.3–15.7%) in different districts in Ghana [14,15].

To control and eliminate SCH and STH, continuous nationwide intervention programs have been instituted by the Ghana Health Service in collaboration with non-governmental organizations (NGOs) for more than a decade in Ghana [16,17]. These interventions have included progressive socio-economic development, health education, and mass drug administration (MDA) with praziquantel (PZQ) and albendazole in various parts of the country [9,18–20]. Although there has been a downward trend in infection over the years, SCH and STH infections are still prevalent in some districts in Ghana [16,21–26]. For MDA to be successful in reducing human SCH and STH, there has to be extensive treatment coverage, compliance with treatment, and the inclusion of both adults and children in the drug delivery coverage in endemic communities [27]. However, in hard-to-reach communities, the sustainability of the MDA campaigns is of concern because these areas are often neglected because they are not easily accessible due to their location. Given that MDA is the primary strategy for SCH and STH control in the country, it is imperative to identify MDA challenges so that targeted strategies to enhance control and improve treatment uptake in infected persons can be achieved. This study aimed to investigate challenges in achieving sustainable coverage of mass drug administration for the control of SCH and STH and to explore opportunities to scale up its implementation among people living in hard-to-reach communities.

## Methods

### Ethics statement

We hereby confirm that all methods were carried out in accordance with relevant guidelines and regulations with the Declaration of Helsinki. Ethical clearance was sought from the Ethics and Protocol Review Committee (EPRC) of the College of Health Sciences, University of Ghana (Ethical approval no: CHS-ET/M.2-4.10/2018-2019). For all study participants, formal verbal consent was sought before involvement in the study.

### Study design and sites

This cross-sectional study was conducted at three sites, two (2) island communities along the Volta River: Twenikorpe (5° 48′ N, 0° 37′ E, 3 m) and Pediatorkope (5° 49′ N, 0° 37′ E, 3 m) in the Ada East District of the Greater Accra Region, and Meterkpor, a town near Akosombo (6.2668° N, 0.0443° E) in the southern part of the Asuogyaman District, Eastern Region, from April to November, 2022. The islands were purposefully selected because they are hard-to-reach by nature, and often accessible only by paddled canoes. Twenikorpe has a population of about 7,000 people, while Pediatorkope is made up of 22 scattered villages and communities with a total population of about 10,000 [28]. The inhabitants of these three (3) sites are mainly engaged in fishing and rural subsistence farming.

### Study procedure and participant selection

This qualitative study utilized focus group discussions (FGDs) and in-depth interview (IDI) methods. In each community, approximately six FGDs were formed, stratified by age, with each group consisting of 10–15 participants. Three FGDs included younger adults (18–35 years), and the other three included older adults (36 years and older). Simple random sampling was used to select the FGD members in each community. Participants were contacted by community volunteers and research team members, and once the study was explained to them and consent was obtained, they were invited to participate. Criteria for the selection of participants were that members of the community must be 18 years of age or older, and they should have lived for at least 5 years in the study sites.

The FGDs included community members such as school teachers, nurses, and opinion leaders from the selected communities. These discussions aimed to investigate the challenges and opportunities in achieving sustainable coverage of MDA (Mass Drug Administration) and how treatment efforts could be optimized for disease control and elimination in their communities.

For the IDIs, key informants such as nurses, teachers, and traditional and other group leaders were visited by the community volunteers and research team. The study was explained to them, and consent was obtained prior to conducting the interviews.

### Data collection

The data collection was moderated by the principal investigator and assisted by two trained field assistants who were trained prior to the start of the survey with the aid of interview guides (**S1 Appendix**). The training covered an overview of the protocol of the project, how to invite study participants, obtain informed consent, administer the interview questionnaire, and practice interview sessions. This structured interview guide was translated into the local dialect (Ewe for Twenikorpe and Dangme for Pediatorkope). Adjustments were made to the questions that needed corrections based on the results of the translation and back-translation.

The design of the study was interactive, and there was a back-and-forth process of questioning during the data collection. Further probing was undertaken to the point where no new information could be obtained from the participants gathered. A major standard procedure that was used was the maintenance of a neutral stance while probing for answers. This ensured the participants were given room to express themselves without leading questions. Each FGD and IDI took a minimum of 40 mins to a maximum of 60 mins, and both were held in private areas to ensure participants’ confidentiality. Notes were taken during the data collection process, and voice recorders were used to record all the information.

The Data collected from the FGDs aimed to assess knowledge and perceptions about SCH and STH, perceptions and attitudes toward MDA interventions, and the challenges of achieving sustainable MDAs for SCH and STH in the community.

Key informants (school teachers, nurses, and community group leaders’) were engaged through in-depth interviews (IDIs) to discuss how treatment efforts could be scaled up or improved to help eliminate SCH and STH. The in-depth interviews also focused on reasons community members refused to adhere or challenges hindering their adherence to administered treatment. Also, questions were asked to assess knowledge and perceptions of MDA and the role of CDDs, as well as perceptions and attitudes towards MDAs. Questions on the control of MDAs to determine the extent of coverage of preventive measures were also asked.

### Data management and analysis

The recorded data were coded and later transcribed and translated into English. Transcripts from recordings were analyzed using the thematic analytical approach described by Attride-Stirling [29]. Quotes that best captured the essence of what was represented were illustrated. Manual analysis was also carried out with the study’s identified themes, which were determined prior to the analyses. A coded sheet was created following the IDIs and FGDs guides, after which the textual data was coded into selected themes and a master sheet analysis was carried out. The themes were: the challenges associated with the implementation and uptake of MDA in hard-to-reach communities in Ghana; and the opportunities that are available to improve MDA for the control and elimination of SCH and STH in hard-to-reach communities in Ghana. These main themes were developed from the sub-themes extracted from the responses. Representative quotes have been embedded within the results to illustrate themes, with minor grammatical alterations to improve readability.

## Results

### Background characteristics of FGD and IDI participants

In total, 12 FGDs with a total of 215 community members and 20 IDIs were conducted at the 3 study sites. The majority of the study participants were males (54.4%) and over 35 years (51.1%) of age. The socio-demographic characteristics of the participants are presented in Table 1.

**Table 1:** Demography of focus group discussion members.

		Gender	
Age Groups	Community	Male No. (%)	Female No. (%)
18–35	Meterkpor	18 (35.3)	15 (51.7)
36–80	Meterkpor	33 (64.7)	14 (48.3)
		51 (100)	29 (100)
18–35	Pediatorkorpe	20 (52.6)	21 (51.2)
36–80	Pediatorkorpe	18 (47.4)	20 (48.8)
		38 (100)	41 (100)
18–35	Twenikorpe	13 (46.4)	19 (67.9)
36–80	Twenikorpe	15 (53.6)	9 (32.1)
		28(100)	28(100)
Total		117 (54.4)	98 (45.6)

In-depth interviews were conducted with 16 respondents from three communities (Pediatorkorpe, Twenikorpe, and Meterkpor). At Meterkpor, eight key informants, including an assemblyman and three teachers, were interviewed. However, at Pediatorkorpe, the Assemblyman (Political community leader), 3 nurses, and 5 teachers were interviewed, and at Twenikorpe, the Assemblyman and two youth group leaders were also interviewed. The participants were purposely selected due to the critical role they play in the community in relation to MDA. The assemblymen are the political leaders in the communities; the teachers usually help in MDA; and the nurses are health professionals in the community.

### Focus group discussions

#### Knowledge and Perceptions of SCH and STH by community members

In all the focus group (FG) discussions with community members and IDIs, it was noted that the residents were more familiar with SCH than STH. The participants demonstrated some knowledge of the symptoms of SCH disease, and causes infections. The respondents also reported that there was no discrimination or stigmatization of people who have SCH or STH. However, infected people were disallowed from sharing things with other family members.

“*They are small animals in the river that enter the body when you fetch water from the river to drink without sieving it. It makes some people’s stomachs bloat, some get fatigued…It doesn’t help the body develop…It also makes some people urinate blood.”* (FG from Meterkpor)

“*There are some worms in the river that cause the diseases*. *People in the community have itchy skin after bathing in the river*……*some also have blood in their urine*.” (Metapor IDIs)

“*SCH is not a major disease in the community compared to malaria*. *Yes*, *I have seen someone affected by schistosomiasis*, *but that was a long time ago*. *I know about STH*. *STH is another disease that affects children and a few farmers because most farmers wear wellington boots to protect themselves*. *I cannot tell when there is an infection with SCH*, *but with malaria*, *I know the symptoms*. *STH are worms in the soil and mostly affect those who have contact with the soil*. *SCH affects those who swim in the river*.”(Pediatorkorpe IDIs)

Community members were aware of MDA activities and proceeded to describe how MDA is carried out in their various communities mainly involved teachers and how administration of the drugs were done.

“*The MDA is done once every two years. The MDA is done by checking their heights before giving them the drugs. The MDA is done in the community. They gather the community members for distribution. They do not move from house to house for the distribution of drugs. Hence, the MDA doesn’t cover all households in the community because there is no prior notice. The drug distributors come to the community, and an announcement is made to gather the members of the community.*” (FG Meterkpor)

“*The mass drug administration is done by gathering the community members at a commonplace in the community*, *and then the distribution is done*. *It is not a house-to-house distribution*. *They are not able to distribute the drugs to the whole community because the drugs are usually in limited supply*. *It is mostly the people who are affected by the disease who go for the drug*. *They use first come*, *first served in the distribution of the drug*. *The MDA exercise is done once a year*.” (Metapor IDIs)

Participants in the community FGDs also reported that the common side effects of the drugs were diarrhea, vomiting, general body weakness, dizziness, and a protruding stomach. Despite these adverse experiences they insisted the MDA should continue since it was proven to be the panacea for SCH and STH control.

“*The MDA has been very useful in the community; people get better after taking the drugs, and those that had bloody stools and urine after taking the drug had no bloody stools and urine.*”(FG from Twanikorpe)

### Challenges reported by community members

The respondents enumerated the following challenges associated with the MDA in their communities:

Limited education and the adverse effects of the drugs, coupled with parental control and some personal cultural beliefs of some community members, lead to the avoidance of MDA.

“*The officials of the MDA do not give room for questions and explanations of the exercise….they do not give any education on the drug and only administer the drugs and leave.*” (FG Meterkpor).

“*The drug given during the MDA weakens the body and also makes the individual sleepy*, *vomit*, *or experience diarrhea when taken*, *and you are unable to go about your activities*, *so it scares some people from taking it*.” (FG Pediatorkorpe)

“*Some of the children refuse to go to school anytime there is an announcement for MDA due to the side effects from the drugs*, *and some refuse to take the drug because of the adverse experiences from others from taking the drugs*.” (FG Meterkpor)

"*There is no education about the SCH and STC infection in the community*, *and so some people refuse to take the drugs*… *some of the children exaggerate about the symptoms and adverse experiences from the drugs*, *so most parents advise their children and neigbours about the adverse experiences of the drugs and tell them not to take the drugs*." (Pediatorkorpe IDIs)

"*I’m not aware of any cultural practice that made them go to the river’s water*. *Some community members believe the treated water has SCH particles in it*, *so they prefer the river water*. *Some people say the river water tastes better*, *so they prefer it to the tap water*." (Pediatorkorpe IDIs)

According to the respondents, MDA happens once a year, and the drugs available for the exercises is limited to the schools or insufficient, thereby depriving the entire community members of access to the drugs.

"*The MDA usually does not benefit the community because the distribution does not cover the whole community, usually due to the drugs being in limited supply.*" (IDI, Meterkpor)

"*The MDA cannot reduce the prevalence of SCH and STH because*, *for now*, *only schoolchildren benefit from the exercise*. *The whole community benefited from the MDA over a decade ago*, *but the schoolchildren were recently given drugs in 2021*.*"* (FG -Pediatorkorpe).

The participants also mentioned lack of CDDs (Community drug distributors) in the communities to create awareness and distribute the drugs was a challenge.

“*We do not have CDDs in this community, and when someone has SCH and STH, they first seek help from a hospital, buy drugs from the drugstore, and some people use herbal medicine for treatment as a last resort*” (FG—Meterkpor)

“*I’m not aware of the community drug distributors for SCH and STH*. ***…*..** *I don’t know about it…*.. *When someone has SCH and STH*, *they go to the health facility in the community*.” (IDD -Pediakorpe)

The participants also echoed that some socio-economic activities (e.g farming, fishing, and oystering) of the indigenes enabled continuous contact with the infected water bodies and exposed them constantly to SCH and STH infections.

“*The people infected mostly in the community are the youth; they mostly fish in the river but do not know whether the disease is from the river or not. The fishermen also said they do not have any choice but to fish in the river for their daily bread.*” (FG—Twenonokorpe)

“*This river is the only water source we have and our source of livelihood; we do not have any other options*.” (FG—Pediakorpe)

Lack of safe means of transportation to affected communities and communities’ festival activities during MDA were also identified as a challenge.

"*Access to some of the communities is a challenge. This is because to get to some communities, one must cross with a canoe, which is quite scary. This challenge can be resolved by providing life jackets and good means of transportation for crossing the river”* (Pediakorpe IDIs)

"*There is some participation in MDA in the community; however*, *participation is very low when there is a festival in the community*." (IDD -Pediakorpe)

### The opportunities available to control SCH and STH

According to the respondents, education was identified as the dominant tool to resolve some of the challenges confronting the MDA and prevent SCH or STH infections in the communities.

"*Other measures that will help reduce SCH and STH in the community are education, and I think if books on SCH and STH are made available for children to read, it will be very helpful. Jingles on SCH and STH should be played on their radio stations……..if educational materials on the disease are made available, it will help. Pictures on SCH/STH should be made available and pasted in the classrooms*." (IDI-Pediakorpe)

“*People should be encouraged to always wear their slippers*, *and there should also be education on the importance of wearing slippers when going to the riverbank*. (FG—Pediakorpe)

An increase in MDA to at least twice a year in the communities and the establishment of CDD in the communities was also echoed by the participants to help control *SCH and STH infections*.

"*Unfortunately, there are no community drug distributors for SCH and STH, and having them in the community will help provide the drugs to affected persons in the community*." (Twenokorpe IDIs)

"*The way the MDA is carried out in the community*, *it cannot help reduce the prevalence of SCH and STH because the MDA is done once a year*, *and the last time drugs were distributed was in November 2021*. *It should be carried out at least twice a year*.” (Pediakorpe IDIs)

Respondents also reported that collaboration with all stakeholders to embrace the initiatives of MDA was identified as a useful tool.

"*The challenge is engagement with stakeholders, because as a community we have pleaded with them to provide us with potable water, but there has been no response. Some stakeholders use the river water for tilapia farming, and a lot of chemicals are released into the river, which is not good for the health of the people in the community. The problem can also partly be blamed on community members who constantly bathe in the river. Another challenge is the attitude of the community members towards the reduction of the disease.*"(Metapor IDIs)

The participants also echoed the need for the provision of after-MDA support for people who may react adversely to the drugs and take measures to avoid contact with the infected river

"*The disease is endemic in the community; since there is no after-MDA support and because there are serious side effects from the drugs, most children stay out of school for a week because of the adverse experiences they encountered from taking the drugs."* (Pediakorpe IDIs)

"*These challenges can be resolved when the community members stop bathing in the river*. *Also*, *if the people boil the river water before using it*, *the provision of concrete slabs for standing on while fetching water from the river can a be good solution*.*"* (Metapor IDIs)

## Discussion

This study explored implementation challenges and opportunities to improve MDA uptake based on the experiences and perceptions of community members and key informants living in hard-to-reach communities in Ghana. The results have shown that there are several challenges experienced by community members during the MDA campaign. Challenges of implementation reported in this study were inadequate education of MDA campaigns, adverse effects of the drugs, coupled with parental control, and personal cultural beliefs of some members leading to avoidance of MDA. From the perspective of community members and key informants, the results also show opportunities to mitigate the challenges that could potentially improve MDA uptake.

Generally, the community members reported a low level of education in SCH and STH, and poor communication of MDA scheduled activities was a major challenge that led to the community members refusal to participate in MDA. The low level of education could be due to the packaging of messages during the delivery of health education for SCH and STH. Similarly, results of studies conducted in Philippines [30] and India [31] reported knowledge gaps about the disease, MDA activities, and the schedule of MDA led to low participation of community members. Education, effective communication, and engagement of community members in SCH and STH prevention and control activities are crucial for improving community members availability during MDA [32]. The education should target informing and enlightening people on the diseases, thus creating awareness of their general knowledge, symptoms, and causes. Once the symptoms are well known and understood, residents will appreciate the presence of SCH and STH in their communities, which will make them value and participate in MDA.

The community members perceptions and public knowledge can motivate the community’s decision to participate in MDA activities despite adverse effects. In this study, past experiences with adverse effects without proper guidance or assistance from MDA providers remained one of the main reasons why members absented themselves from such initiatives at schools or in the community, even when the drugs were free. This study recommends implementing a context-adapted community engagement strategy that leverages existing community structures and takes into consideration past community experiences of MDAs to control STH and SCH. Similar **s**tudies from southern Malawi, Tanzania, and Uganda revealed similar sentiments and adverse effects of the drugs, which led to community members disallowing their children or absenting themselves from MDA activities [33–35]. The importance of delivering appropriate information to enable awareness about the SCH and STH and making arrangements for the management of side effects from drugs administered during MDA has been reiterated in a study conducted in West Bengal, India [36].

The insufficiency of drugs issued to community members was another challenge to the implementation of MDA. Senyonjo *et*. *al*. [37] reported drug shortages in Cameroon were attributed to the failure of the MDA program. Further, some community members expressed concern that children were prioritized over adults, although both SCH and STH were endemic in all age groups in the communities. An appropriate drug delivery strategy to reach all community members is highly relevant to improving MDA uptake and aiding the control of STH and SCH [38,39]. Similar studies in Turkey [40] and the Philippines [41] have reported health systems prioritizing children over adults during MDA activities.

Based on the current study results, the lack of CDDs in the communities was reported to be a challenge to the implementation of MDA. CDDs are very important for community census, sensitization, and drug distribution [38]. The Ghanian NTD program has been running for many years [3], and to ensure proper involvement of the communities in MDA, the current study recommends the NTD program consider selecting and training CDDs in the communities to ensure quality campaigns and high treatment uptake for SCH and STH. In the Island communities, due to a lack of CDDs, school teachers participated and helped execute the MDA in schools. However, the teachers are not equipped to manage adverse effects associated with the drug, and parents felt the teachers were not adequately trained to administer the drugs. However, a study conducted in Nigeria expressed the opposite opinion, where parents preferred school teachers to execute the drug administration since they were closer to the pupils [42].

The study revealed there were no superstitious beliefs or misconceptions that led to the stigmatization of people living with SCH and STH, nor were there any cultural or mystical beliefs about the disease. However, some cultural behaviors were reported to pose a challenge to SCH and STH control. Walking barefoot was reported to be an unconsciously acceptable behavior among children, and bathing in the river was a common practice in the village. This is in contrast to the Musuva *et al*. [43] study in Kenya, which reported that misconceptions about SCH led to stigmatization and was a barrier to schistosomiasis control. The respondents also reported that although SCH and STH were endemic in the community, the diseases were not given health priority in the community compared to other acute health problems such as malaria. This suggests that community members are not well informed about the disease, consistent with findings from Kenya [44]. Control and eventual elimination of SCH and STH could be very difficult if affected communities do not perceive the disease as a major public health problem. It is imperative to developing an efficient strategy with emphasis on behavioral change to sufficiently raise disease awareness to a level that influences treatment-seeking behavior and preventive practices. A similar study in Tanzania by Angelo *et al*.,[45] reported that community members gave major priority to malaria and cholera compared to SCH because they believed they were more acute and life threatening.

## Conclusion

The findings of this study revealed that there are several implementation challenges experienced by community members that are related to socio-economic, cultural, and health systems, as well as the MDA program itself, that should be addressed in a more holistic way. Participants recognize these gaps, leading to vocal requests for better information dissemination to deal with prevailing misconceptions and fears about SCH and STH treatment, adverse effects, and negative cultural behaviors. To control SCH and STH infections, political members and policy makers must collaborate to provide scarce infrastructure, build and renovate sanitary infrastructure in hard-to-reach communities to support intellectual activities and ensure hygiene in the communities. Furthermore, to completely eliminate the barriers and challenges that obstruct MDA operations in places that are difficult to access, policymakers and program implementors should work with the community to establish and implement a comprehensive practical disease control plan that includes both extensive community education and support for MDA operations.

## Supporting information

S1 Appendix Interview Guides for (A) Focus Group Discussion (B) Individual Interview Guide for key informants. (DOCX)
