# Supplementary material for: Knowledge, Perceptions, Challenges and opportunities in achieving sustainable coverage of mass drug administration towards the control and elimination of Schistosomiasis and Soil Transmitted Helminths in hard-to-reach communities of Ghana
Source: PLoS Negl Trop Dis. 2024 Nov 21;18(11):e0012664. doi: 10.1371/journal.pntd.0012664 (PMC11620658; doi:10.1371/journal.pntd.0012664)
Supplement: S1 Appendix — (DOCX) [file pntd.0012664.s001.docx]

**Appendix 1 - Interview Guides**

**A. Focus Group Discussion Guide (Community members)**

**Socio-Demographic Characteristics**

Name of Region:……………………………………… Moderator:

Name of Community:………………………………. Notetaker:

Date of FGD:……………………………………………. No of Participants:

Location of FGD:…………………………………….. Start time…………

Could each of you tell me about yourself? Can each of you tell me about yourself?

*Probe: Age, education, occupation, length of stay in community*

**Knowledge and perceptions on schistosomiasis (SCH) and Soil Transmitted Helminths (STH) and role of CDDs.**

1. Do you know about SCH and STH, if yes, do tell us what you know
2. What is the source of transmission of SCH and STH?

Probe: Air, water, soil or food.

1. Is SCH and STH a major health problem in this community?
   1. If YES why?
   2. If No why

*Probe: Is any member of your family having schistosomiasis or STH, and if they have had it in the last two years?*

1. Which people are the mostly affected in your community and why? *Probe: Children? Girls? Women?*
2. What can be done to prevent SCH and STH in this community?
3. Are you aware of the community drug distributors for SCH and STH and their role?
   1. Probe for the role of the CDD from participants
4. When someone has SCH and STH, where do they first seek help from and why?

*Probes. a) Home, health facility, church, CDD, herbalist*

*b) Where do they go for ultimate care and why?*

1. Could you describe to me what you know about how mass drug administration is done in this community?

*Probe: a) How often is MDA undertaken in this community?*

*b) Are all households in the community involved or covered?*

**Perceptions and attitudes on interventions**

1. What do you think are the benefits or perceived impacts of the MDA?
2. Do you know of any negative effects of the MDA drugs? If yes, explain
3. Are there people in this community who refuse to take the drugs,
   1. If YES why?
4. Considering the way the MDA is carried out in this community, can it help to reduce the prevalence of SCH and STH?

Probe: a) Why or why not?

b) If yes, what are the positives things about the way the MDA is carried out?

c) If no, what do you think could be done to improve the implementation?

**Religious, Cultural and Socioeconomic issues**

1. Do people who get schistosomiasis in this community face discrimination and/or stigma? If yes, explain.

17. If you (participants) know that it is the (water/soil) why do community member keep going back to the water or walking bare foot on the soil?

Probe? Cultural or traditional reason

18. Would you like another source of water for your community?

Probe: for suggestion- If yes, why? If no, why?

19. Are there any other social amenities that if your community is provided would help solve schistosomiasis or soil transmitted Helminths transmission in your communities?

Probe? For suggestions and why that specific suggestion.

19. Are there any religious or cultural believes that may be preventing people from taking the drug?

Probe? For other reasons and why?

21. Are there any religious, cultural and/ traditional practices that keeps taking the community members to the water? Probe? For reasons and why?

20. Are there people (elderly) in this community who refuse to take the drugs, and why?

**Challenges and opportunities for achieving sustainable MDAs**

1. Have people reported any problems after taking the MDA drugs?

Probe: What are the complaints about the drugs, the exact problem reported, how the problem was managed and who managed the problem etc.

1. As beneficiaries of the intervention (MDAs), what will you say are some of the challenges?
2. What issues if ANY do you have as community members towards MDAs?

*Probe: Attitude, work ethic etc*

1. How can these challenges be resolved? *Probe further*

**CDDs challenges to help retention and sustainability**

1. In your opinion, do CDDs to work effectively to distribute the drugs?

Probe: If yes, why? If No, why?

1. What do you think should be provided to support CDDs to work effectively in the community?

Probes: Drugs, means of transportation, umbrellas, incentives

***Possible solutions to avoid contact with SCH in the water***

1. Are you aware that storing water from the river for some days before usage can help kill the SCH parasite?
2. If the community is provided with storage tanks to store the water for some days before you have access to it, will you be happy to use it.

Probe: If yes why? If no, why?

1. If the community was provided with slaps for standing on while fetching water from the river or washing, will you be happy to use it?

Probe: If yes why? If no, why?

**Possible solutions to avoid contact with STH**

1. Can you please give suggestions on how community members can be encouraged to ware foot wares so as to avoid contacts with STH?
2. Aside foot wares what are the measures that could be used to control STH in your community? Probe further.
3. Have anyone here ever had any health education on STH and how to avoid getting infected?

Probe: when and how?

**Sustainability of MDA**

1. What are the factors or challenges affecting MDA?

Probe: inadequate of basic needs for work, attitude of community members, health systems challenges, bottlenecks etc.

In our view how can these challenges you have mentioned be resolved? Probe further for answers.

1. Considering the current MDA implementation, can it help to reduce the prevalence of schistosomiasis and STH in your community?

26. If yes, what are the positives that could be sustained? Probe further for answers.

27. If no, what do you think could be done to improve the implementation? Probe further for answers?

28. Any questions or comments that you would like us to know about?

**B. Individual Interview Guide (Key informants)**

**For House hold leaders, Community Group leaders, District Health officer**

Can you tell me about yourself?

Probe: Age, education, occupation, length of stay as DHO

**Knowledge on MDA and role of CDDs**

1. Could you please tell me anything you know about schistosomiasis (SCH) and Soil Transmitted Helminths (STH) in this community?
2. Are they a big problem in this community?

Probe why or why NOT?

1. Who are the most affected in this community and why?

Probe: Children? Girls? Boys? Women?

1. In your view what can be done to prevent SCH and STH in this community/district?
2. Could you describe what and how mass drug administration is done in your community/district?

Probe: a) How often is this done?

b) Are all households in the district involved or captured?

1. Do people with SCH get discriminated and stigmatized in your district?

Probe: If yes, explain

**Perceptions and Attitudes on Interventions**

1. What do you think are the benefits or perceived impacts of the MDA?
2. Do you know of any adverse effects from using the drugs? If yes, explain
3. Considering the current MDA implementation, can it help to reduce the prevalence of SCH and STH in the community?
4. What are some of the positives things about the MDA that could be sustained?

What can be done to improve the implementation of the MDA in the district?

1. When was the last time MDA for SCH and STH was undertaken in the community?

Probe about times of administration if answer is yes, if non, reasons they were not being carried out

1. How was the participation of your community members in the last MDA for SCH and STH in the community? Probe for any problems or challenges
2. How can drug distribution in your community be improved? Probe about method of distribution, drug distributors and duration of duration
3. Do community member know what the sources for SCH and STH are?
4. If community members know it is the water or the soil, why do they keep going back into the water and/ walking bare foot on the soil surfaces

Probe: domestic activities – fetching water for cooking, swimming, washing, fishing etc

1. Are there any other social amenities that the community need to help reduce the transmission of schistosomiasis or soil transmitted Helminths in your community or district.

**Religious and Cultural Perceptions**

1. Are there any religious believe that may be preventing people from taking the drug in this community/district?

Probe? From the reason and any religious known religious believes

1. Are there any cultural and traditional practises that keeps taking community members back to the water?

Probe? answers

**Health system challenges**

1. What are the health system challenges that is preventing CDDs from working effectively in your community/ district if there are any?

Probe: Financial, training or capacity building, supplies and logistics, morale booster etc.

20. What kind of support do CDDs receive from your district health facility? Probe: financial, moral, food, others?

21. Is this support adequate for SCH or STH control and elimination?

Probe: If yes, Why? If no, why?

**Sustainability of MDA**

22. Are all sections of the community/district covered by the drug distribution? If not why?

23. What are the factors or challenges affecting MDA in your district?

Probe: inadequate of basic needs for work, attitude of community members, bottlenecks etc.

24. In your view how can these challenges be resolved? Probe further for answers

25. Considering the current MDA implementation, can it help to reduce the prevalence of schistosomiasis and STH?

26. If yes, what are the positives that could be sustained? Probe further for answers.

27. If no, what do you think could be done to improve the implementation? Probe further for answers.

28. Any questions or comments that you would like us to know about?
